# Sleep-timing irregularity moderates the within-participant association between training load and next-morning heart rate variability in university sport participants: a 28-day study

**DOI:** 10.3389/fphys.2026.1966561

**Published:** 2026-09-23

**Authors:** Xiangyu Wang, Yongwei Wang

**Affiliations:** Department of Physical Education, Capital Normal University, Beijing, China

**Keywords:** athlete monitoring, heart rate variability, intensive longitudinal data, RMSSD, sleep midpoint, sleep regularity, training load

## Abstract

**Background:**

Heart rate variability (HRV) describes beat-to-beat variation in cardiac intervals; the root mean square of successive differences (RMSSD) is a time-domain index of short-term cardiac vagal modulation. We examined whether nightly deviation from habitual sleep midpoint moderated the association between prior-day internal training load and next-morning natural-log-transformed RMSSD (lnRMSSD), and whether analogous interactions were present for resting heart rate, wellness strain, and countermovement-jump (CMJ) height.

**Methods:**

University students engaged in regular sport training completed 28 days of habitual monitoring. Internal load was session duration multiplied by session rating of perceived exertion. Wrist actigraphy and sleep diaries estimated sleep midpoint, and 5-min morning RR recordings yielded lnRMSSD. Mixed models separated within- and between-participant components and tested within-participant load (per 100 arbitrary units [AU]) × absolute midpoint deviation (per 30 min).

**Results:**

The analysis included 1,232 mornings from 54 participants (57.4% female; age 21.0 ± 1.7 years). At each participant’s mean midpoint deviation, greater-than-usual load was associated with lower next-morning lnRMSSD (β = -0.0089, 95% CI -0.0127 to -0.0051; p < 0.001). At mean load, greater-than-usual midpoint deviation was also associated with lower lnRMSSD (β = -0.0156, 95% CI -0.0268 to -0.0045; p = 0.006). The interaction was negative (β = -0.0101, 95% CI -0.0153 to -0.0048; p < 0.001). Thus, the inverse within-participant association between load and lnRMSSD strengthened as sleep-timing irregularity increased. No load-by-midpoint interaction was detected for resting heart rate, wellness strain, or CMJ height after false discovery rate adjustment. The primary interaction persisted after adjustment for prior-morning lnRMSSD (β = -0.0096, 95% CI -0.0147 to -0.0046; p < 0.001).

**Conclusion:**

The within-participant association between training load and next-morning cardiac vagal modulation was moderated by sleep-timing irregularity. These field-based associations do not establish a sleep mechanism, causal recovery effect, or decision threshold.

## 1 Introduction

Athlete monitoring is most informative when prescribed work and the athlete’s response are interpreted together. Internal training load is commonly estimated as session rating of perceived exertion (sRPE) multiplied by session duration, a practical measure that can be used across training modes (Foster et al., 2001; Impellizzeri et al., 2004; Bourdon et al., 2017). Morning heart rate variability (HRV), the fluctuation in intervals between successive heartbeats, is likewise used to characterize cardiac autonomic modulation in sport (Task Force, 1996; Buchheit, 2014; Lundstrom et al., 2023). The root mean square of successive differences between RR intervals (RMSSD) is a time-domain index that predominantly reflects short-term cardiac vagal modulation; its natural logarithm (lnRMSSD) is often analyzed to reduce positive skew and stabilize variance (Shaffer and Ginsberg, 2017; Laborde et al., 2017). Within a standardized morning context, a lower-than-usual lnRMSSD denotes lower vagally mediated beat-to-beat variation that morning. No single marker captures fatigue or adaptation, so recent training and recovery context remains essential (Halson, 2014). It does not, by itself, identify poor adaptation: both increases and decreases in vagal indices may occur during positive adaptation (performance improvement) or negative adaptation (fatigue, overreaching, or performance decrement), depending on training phase and the athlete’s recent trajectory (Plews et al., 2013; Bellenger et al., 2016). Because HRV interpretation depends on this context, identifying reliable within-participant moderators such as sleep-timing irregularity can improve interpretation independently of whether a given HRV change is ultimately judged adaptive or maladaptive.

Free-living monitoring refers here to measurements collected during habitual, unsupervised daily life rather than in a laboratory intervention. In a large free-living dataset, acute training was followed by small average changes in resting heart rate (HR) and HRV, whereas alcohol intake, illness, and menstrual-cycle phase produced different response profiles (Altini and Plews, 2021). Elite-rower records likewise showed that day-to-day HRV patterns were highly athlete-specific, cautioning against universal thresholds (Plews et al., 2017). A recent 12-week endurance-athlete cohort jointly monitored load, HRV, fatigue, and sleep but focused on injury patterns rather than the day-level sleep-timing interaction tested here (Sanchez et al., 2025). These observations support within-person analysis and contextual interpretation rather than treating a workload or HRV value as self-explanatory.

Sleep may provide part of that context. Athlete sleep is influenced by training and competition schedules, travel, academic and psychosocial demands, and individual sleep need (Fullagar et al., 2015; Walsh et al., 2021). Experimental sleep loss can impair selected physiological, cognitive, and performance outcomes, but responses differ across outcomes and individuals (Fullagar et al., 2015; Skein and Hartmann, 2026). A meta-analysis of 11 randomized controlled trials involving 549 participants found that sleep deprivation reduced RMSSD, whereas changes in the standard deviation of normal-to-normal intervals (SDNN) and high-frequency power were not statistically significant, indicating that autonomic responses varied across HRV indices (Zhang et al., 2025). In 56 junior endurance athletes monitored for 61 days, higher mental strain preceded shorter or less efficient sleep, training load preceded less rapid eye movement sleep, and several sleep dimensions predicted lower subsequent self-selected training load, demonstrating reciprocal temporal associations (Hrozanova et al., 2020). Similarly, in 65 Division I collegiate football players monitored for 3 weeks, within-person variation in sleep latency and efficiency predicted next-day maximum acceleration, and daily acceleration predicted subsequent sleep, whereas between-person effects were not detected (Kilwein et al., 2026). These findings support day-level, within-person analysis of reciprocal associations between sleep and training or performance in university sport participants. Night-to-night variability is therefore a substantive dimension of sleep behavior: it quantifies departures from an individual’s typical pattern rather than only the person’s average sleep (Bei et al., 2016; Kemp et al., 2023).

Sleep timing is particularly relevant when HRV is obtained during a morning routine. Irregular sleep–wake timing has been associated with delayed circadian timing and poorer academic performance in undergraduates and with lower endothelial function in young adults (Phillips et al., 2017; Nakashima et al., 2022). Time of day and chronotype can influence autonomic measurements and exercise responses (Vitale et al., 2019). In female soccer players, training time and match location were associated with sleep timing and nocturnal cardiac autonomic activity, while tournament monitoring showed substantial within-player variation in sleep and HRV (Costa et al., 2019a, 2019b). Total sleep time measures sleep quantity, whereas midpoint deviation captures whether the sleep episode occurred at an atypical time for the same person. We therefore selected absolute midpoint deviation as the primary moderator and adjusted for sleep duration and efficiency in sensitivity analysis rather than treating these non-equivalent dimensions as substitutes.

The primary aim was to determine whether within-participant absolute sleep-midpoint deviation moderated the association between prior-day internal training load and next-morning lnRMSSD. We hypothesized that the association between greater-than-usual load and lower lnRMSSD would be more negative after nights with greater-than-usual midpoint deviation. Secondary aims tested whether the same interaction pattern was present for resting heart rate, a study-specific wellness-strain composite, and countermovement-jump (CMJ) height, which represent distinct cardiovascular, perceptual, and neuromuscular field indicators. Because all exposures were observed rather than assigned, the estimands were interpreted as associations among practical monitoring indicators, not as causal effects or direct measures of sleep physiology, recovery mechanisms, or training adaptation.

## 2 Materials and methods

### 2.1 Study design and participants

This prospective intensive longitudinal observational study was conducted at Capital University of Physical Education and Sports, Beijing, China. Participants were recruited by telephone contact, campus posters, and notices distributed through WeChat, a mobile messaging and social media platform widely used in China. Monitoring took place from 1 to 28 November 2022. Eligible participants were 18–25 years old, were enrolled at a university, had engaged in regular structured sport training at least three times per week for at least 6 months, and could follow the morning measurement procedures. For eligibility, structured sport training was operationalized by these prespecified frequency and training-history thresholds; formal varsity-team membership or competition level was not required. The sample is therefore interpreted as university students engaged in regular sport training, not as an elite or varsity-only cohort. Exclusion criteria were a current injury or acute illness that prevented habitual training, known cardiovascular disease, a diagnosed sleep disorder, medication that materially affected cardiac autonomic function, or inability to complete the 28-day schedule.

At baseline, participants reported age, sex, primary sport category, weekly training frequency, and years of structured sport training; stature and body mass were measured. The primary sport was classified by its predominant demands as endurance, team, or strength–power. Training history denotes the reported number of years of regular structured sport training and was stored to 0.1 year. Eighty people were assessed for eligibility. Twenty were excluded before monitoring because training frequency or history was below the eligibility threshold (n = 7), injury or acute illness affected habitual training (n = 4), a cardiovascular or sleep condition or medication could affect autonomic function (n = 3), or the monitoring schedule could not be completed (n = 6). Sixty entered monitoring. The target sample was determined based on feasibility and capacity to supervise repeated field measurements, without treating daily records as independent participants.

### 2.2 Monitoring schedule

Participants wore the actigraph for 28 consecutive nights and completed an electronic sleep diary. Training duration and category-ratio-10 (CR-10) sRPE were entered approximately 30 min after each session; rest days were recorded explicitly. Each morning, participants completed a seated HRV recording and a brief wellness questionnaire. Three mornings per week also included CMJ testing. Automated reminders were sent through WeChat, and device timestamps and completion records were reviewed daily.

### 2.3 Training-load exposure

Approximately 30 min after training, participants rated the overall exertion of the entire session using a single category-ratio-10 (CR-10) response from 0 (rest/no exertion) to 10 (maximal exertion). Session RPE is intended to integrate the perceived intensity of the whole session rather than the difficulty of a single exercise (Foster et al., 2001; Impellizzeri et al., 2004). Internal training load was calculated as session duration in minutes × session RPE; multiple sessions were summed within each day, and rest days were coded zero. The product was rounded to the nearest whole arbitrary unit (AU). AU is a dimensionless reporting unit for the product, and “whole” indicates integer rounding. The prior calendar day’s total was linked to the subsequent morning and scaled per 100 AU for interpretable coefficients. Load was decomposed into a participant mean (between-participant component) and each day’s deviation from that mean (within-participant component).

### 2.4 Sleep measurement and timing regularity

Sleep–wake behavior was estimated using a wGT3X-BT actigraph (ActiGraph LLC, Pensacola, FL, USA) on the non-dominant wrist. Devices were initialized at 30 Hz; data were aggregated into 60-s epochs and scored in ActiLife 6.13.4 with the Cole–Kripke adult algorithm (Cole et al., 1992). The main sleep interval was first identified from ActiLife output, the electronic diary, and the device event marker. When diary and marker boundaries differed by more than 30 min, two trained assessors integrated these sources with the activity trace and reached consensus. The sleep-onset and final-awakening values used in analysis were the resulting investigator-corrected boundaries, not values from an unreviewed single source. A night was invalid when the interval could not be reconciled or device non-wear precluded scoring.

ActiLife estimated sleep minutes within the scored main interval (total sleep time). Sleep duration in the analysis corresponds to that estimate and can be reproduced as time in bed × sleep efficiency, where time in bed is the elapsed time from sleep onset to final awakening and sleep efficiency is estimated sleep minutes divided by time in bed × 100. Actigraphy is a practical behavioral estimate rather than a criterion measure of sleep physiology and can overestimate sleep or underestimate wake compared with polysomnography (Conley et al., 2019; Halson, 2019).

Sleep midpoint was the circular-time midpoint between sleep onset and final awakening. Habitual midpoint was the circular mean across all valid nights for each participant. Absolute midpoint deviation was the shortest circular distance between each nightly midpoint and that participant’s habitual midpoint. This value was person-mean centered and divided by 30 min. Thirty minutes was prespecified as a readily interpretable, plausible night-to-night shift; rescaling changes coefficient units but not model fit, test statistics, or p-values. Larger within-participant values represent a sleep episode farther from that participant’s 28-day mean timing, irrespective of whether the shift was earlier or later.

### 2.5 Morning HRV and secondary outcomes

Morning RR intervals were obtained with a Polar H10 chest sensor (Polar Electro Oy, Kempele, Finland) paired by Bluetooth with Elite HRV 5.2.1. RR is the elapsed time between successive detected R waves. Participants sat quietly for 5 min and then completed a 5-min recording during the standardized morning sequence while breathing spontaneously and before caffeine, food, or exercise. The protocol standardized posture, rest, duration, and premeasurement behavior, but did not impose a fixed clock time or fixed awakening-to-recording interval; these timings were not prespecified analytic variables. Polar H10 RR intervals show close agreement with electrocardiographic reference recordings at rest (Gilgen-Ammann et al., 2019; Schaffarczyk et al., 2022).

RR intervals were exported and visually reviewed in Kubios HRV Standard 3.5.0 (Kubios Oy, Kuopio, Finland). Threshold-based artifact correction was set to medium (0.25 s), with cubic-spline interpolation of identified intervals (Tarvainen et al., 2014). A recording was valid when at least 240 s of analyzable data remained and no more than 5% of intervals were corrected. RMSSD was calculated as the square root of the mean squared difference between consecutive corrected RR intervals, RMSSD = √mean[(RRi+1 − RRi)²]. RMSSD is a standard time-domain index of short-term vagally mediated HRV (Task Force, 1996; Shaffer and Ginsberg, 2017; Laborde et al., 2017). It was natural-log-transformed before modeling to reduce right skew and stabilize residual variance.

Resting heart rate was taken from the same accepted recording. The morning wellness items assessed current fatigue, muscle soreness, perceived stress, and mood on 1–5 response scales. Higher values indicated worse fatigue, soreness, and stress, whereas higher mood indicated better mood. A study-specific wellness-strain score was constructed as fatigue + soreness + stress + (6 − mood), yielding 4–20 points, with higher scores indicating poorer perceived status. These domains are common in athlete self-report monitoring (McLean et al., 2010; Saw et al., 2016; Duignan et al., 2020), but the four-item sum was constructed for this study and was not treated as an independently validated unidimensional instrument.

CMJ height was assessed with My Jump 2 version 6.1.5 on an iPhone 13 Pro (Apple Inc., Cupertino, CA, USA) recording 1080p video at 240 frames/s. The phone was fixed on a tripod 0.30 m above the floor and approximately 1.5 m from the participant in the sagittal plane. Participants completed three hands-on-hips trials on the same indoor surface with 60 s of rest; the same assessor selected take-off and landing frames, and the best valid height was retained. Trials were invalid if the feet left the camera field, the hands left the hips, or frame selection was ambiguous. Smartphone flight-time methods show generally good validity and reliability for jump height when acquisition is standardized, although manual frame selection remains a source of uncertainty (Balsalobre-Fernández et al., 2015; Vieira et al., 2023; Gençoğlu et al., 2023). Acutely ill mornings were excluded from the primary analysis.

### 2.6 Statistical analysis

Participants required at least 20 valid paired sleep-to-next-morning HRV observations. Descriptive statistics are mean ± SD, median (interquartile range), or number (percentage). The primary linear mixed model used next-morning lnRMSSD as the outcome. Fixed effects were within-participant prior-day load, within-participant absolute midpoint deviation, their interaction, participant means of both exposures, study day, sex (female participants vs. male participants [reference]), training history, and day of week. A participant random intercept and random slope for within-participant load were retained. The interaction estimates the change in the within-participant load–lnRMSSD slope for each additional 30 min by which absolute midpoint deviation exceeded the participant’s own mean. The load main effect is therefore conditional on mean absolute midpoint deviation, and the midpoint main effect is conditional on mean load. Simple load slopes were estimated at the 25th, 50th, and 75th percentiles of centered midpoint deviation. The standardized product-term coefficient was b(interaction) × SD(product term)/SD(lnRMSSD); the raw coefficient was primary.

Models with the same fixed effects were fitted for resting heart rate, wellness strain, and CMJ height; a random load slope was attempted first and simplified only if required. The Benjamini–Hochberg procedure controlled false discovery across the three secondary interaction tests. Generalized estimating equations (GEE) are population-average regression models for correlated repeated observations (Liang and Zeger, 1986). Autoregressive GEE was used to evaluate sensitivity to within-participant serial correlation. Other prespecified sensitivity models addressed adherence (≥24 valid pairs), late-ending sessions (>22:00), sleep quantity and quality (within- and between-participant sleep duration and efficiency), random-effects complexity (random intercept only), and observation availability (unweighted and inverse-probability-weighted independence GEE). Availability weights were estimated from weekend, study day, load, midpoint deviation, and sex; probabilities were bounded to 0.15–0.99.

Additional sensitivity analyses adjusted the primary model for within- and between-participant prior-morning lnRMSSD, adjusted for primary sport category, and replaced absolute deviation with signed circular midpoint shift (positive = later, negative = earlier), person-mean centered per 30 min. These models addressed temporal confounding by prior autonomic status, broad sport-type differences, and potential differences in moderation between earlier and later timing shifts, respectively. Residual distribution, residual-fitted association, optimizer convergence, boundary warnings, random-effects covariance, and within-participant autocorrelation were examined. Mixed-model confidence intervals and p-values were calculated using large-sample normal approximations; GEE inference used participant-cluster robust covariance. Two-sided p < 0.05 defined statistical significance for the single primary interaction, with estimates and uncertainty prioritized. Analyses were conducted in Python using pandas, NumPy, SciPy, statsmodels, Matplotlib, and seaborn; the computational environment is specified in the accompanying analysis files.

## 3 Results

### 3.1 Participant flow and descriptive characteristics

Of 80 people assessed for eligibility, 20 were excluded and 60 entered the 28-day study. Six did not meet the primary-analysis threshold: three discontinued monitoring and three completed monitoring but contributed fewer than 20 valid sleep–HRV pairs. The remaining 54 participants contributed 1,232 observations. The cohort was 57.4% female, had a mean age of 21.0 ± 1.7 years, and reported 4.2 ± 2.0 years of structured sport training. Primary sport categories were endurance (n = 15), team (n = 21), and strength–power (n = 18). These categories describe the predominant demands of the reported primary sport; competition level and formal team membership were not selection criteria. Median valid paired monitoring time was 23 days (interquartile range 22–24; **Table 1**).

**Table 1:** Participant characteristics and training exposure in the primary-analysis cohort (n = 54).

Characteristic	Value
Age, years	21.0 (1.7)
Female participants, n (%)	31 (57.4)
Height, cm	172.6 (8.4)
Body mass, kg	64.0 (8.5)
Structured sport-training history, years	4.2 (2.0)
Endurance/team/strength–power, n	15/21/18
Training days, n / days with available training records (%)	1,016/1,509 (67.3)
Training days per week	4.71 (0.57)
Session duration on training days, min	77.9 (24.2)
Valid paired days, median (IQR)	23 (22–24)

Training records were available for 1,509 of 1,512 monitored participant-days. Weekly training frequency was calculated for each participant as the proportion of available records indicating training multiplied by 7.

Training occurred on 1,016 of 1,509 participant-days with available training records (67.3%); training records were unavailable for 3 of the 1,512 monitored participant-days. Participants completed 4.71 ± 0.57 training days per week on average; training sessions on training days lasted 77.9 ± 24.2 min. Across eligible monitoring days, mean prior-day internal load was 251.5 ± 223.0 AU, sleep duration was 7.35 ± 0.75 h, sleep efficiency was 88.22 ± 3.50%, and RMSSD was 59.51 ± 13.32 ms. Absolute sleep-midpoint deviation had a median of 21 min (interquartile range 10–37).

### 3.2 Primary outcome

At a participant’s mean absolute midpoint deviation, greater-than-usual prior-day load was associated with lower next-morning lnRMSSD (β = -0.0089 per 100 AU, 95% CI -0.0127 to -0.0051; p < 0.001). At mean load, greater-than-usual absolute midpoint deviation was also associated with lower lnRMSSD (β = -0.0156 per 30 min, 95% CI -0.0268 to -0.0045; p = 0.006). The hypothesized within-participant load-by-midpoint-deviation interaction was negative (β = -0.0101 per 100 AU × 30 min, 95% CI -0.0153 to -0.0048; p < 0.001; standardized product-term coefficient = -0.059; **Table 2**; **Figure 1**). Between participants, mean load (β = 0.0034, 95% CI -0.1160 to 0.1227; p = 0.956) and mean absolute midpoint deviation (β = 0.2493, 95% CI -0.1041 to 0.6027; p = 0.167) were not associated with mean lnRMSSD.

**Table 2:** Primary mixed-model estimates for next-morning lnRMSSD.

Term	Estimate	SE	95% CI	P-value	Standardized estimate
Within-participant load, per 100 AU	-0.0089	0.0020	-0.0127 to -0.0051	< 0.001	-0.085
Within-participant midpoint deviation, per 30 min	-0.0156	0.0057	-0.0268 to -0.0045	0.006	-0.042
Load × midpoint deviation	-0.0101	0.0027	-0.0153 to -0.0048	< 0.001	-0.059
Between-participant mean load, per 100 AU	0.0034	0.0609	-0.1160 to 0.1227	0.956	—
Between-participant mean midpoint deviation, per 30 min	0.2493	0.1803	-0.1041 to 0.6027	0.167	—

The model included study day, sex, training history, day of week, a participant random intercept, and a random load slope. AU, arbitrary units; lnRMSSD, natural logarithm of the root mean square of successive differences.

**Figure 1:**
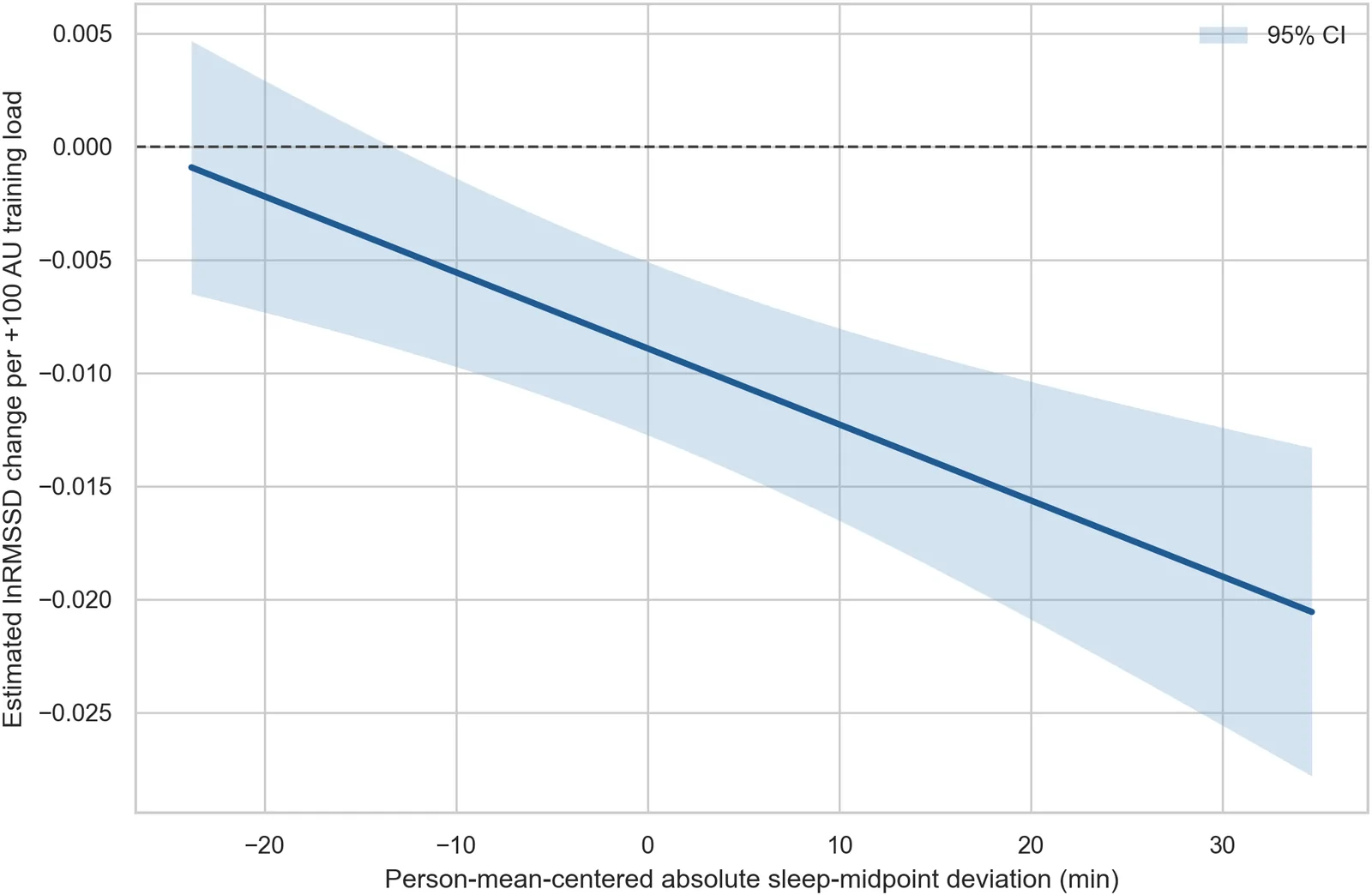
Continuous marginal effect of person-mean-centered absolute sleep-midpoint deviation on the estimated within-participant association between prior-day internal load and next-morning lnRMSSD. The solid line is the model-estimated lnRMSSD change per +100 AU training load; shading is the 95% confidence interval. The dashed horizontal line denotes a zero load slope. The plot spans the central observed moderator range used for estimation.

The estimated load slope was -0.0040 at the 25th percentile of centered midpoint deviation (-14.7 min relative to the participant’s mean absolute deviation; 95% CI -0.0085 to 0.0006; p = 0.085), -0.0077 at the median (-3.7 min; 95% CI -0.0115 to -0.0038; p < 0.001), and -0.0125 at the 75th percentile (+10.8 min; 95% CI -0.0169 to -0.0082; p < 0.001). **Figure 1** displays the estimated load slope continuously across the observed moderator range with its 95% CI.

### 3.3 Secondary outcomes

Greater-than-usual load was associated with higher resting heart rate (β = 0.370 beats/min per 100 AU, 95% CI 0.302 to 0.439; p < 0.001), higher wellness strain (β = 0.498 points, 95% CI 0.453 to 0.544; p < 0.001), and lower CMJ height (β = -0.293 cm, 95% CI -0.329 to -0.257; p < 0.001). Greater-than-usual midpoint deviation was also associated with higher resting heart rate (β = 0.580 beats/min per 30 min, 95% CI 0.361 to 0.799; p < 0.001), higher wellness strain (β = 0.287 points, 95% CI 0.133 to 0.442; p < 0.001), and lower CMJ height (β = -0.228 cm, 95% CI -0.354 to -0.102; p < 0.001).

There were no load-by-midpoint-deviation interaction effects for resting heart rate (β = 0.094, 95% CI -0.009 to 0.196; raw p = 0.073; false discovery rate (FDR)-adjusted p = 0.109), wellness strain (β = 0.067, 95% CI -0.005 to 0.138; raw p = 0.067; FDR-adjusted p = 0.109), or CMJ height (β = -0.038, 95% CI -0.097 to 0.020; raw and FDR-adjusted p = 0.199; **Table 3**). Between-participant mean load was not associated with any secondary outcome (all p ≥ 0.115). Mean midpoint deviation was associated with lower mean wellness strain (β = -1.192, 95% CI -1.904 to -0.481; p = 0.001), but not resting heart rate or CMJ height (p = 0.349 and p = 0.083, respectively).

**Table 3:** Within- and between-participant estimates for secondary outcomes.

Outcome	Effect	Estimate	95% CI	Raw p	FDR p*
Resting heart rate	Within load	0.370	0.302 to 0.439	< 0.001	—
Resting heart rate	Within midpoint deviation	0.580	0.361 to 0.799	< 0.001	—
Resting heart rate	Interaction	0.094	-0.009 to 0.196	0.073	0.109
Resting heart rate	Between mean load	0.276	-0.818 to 1.369	0.621	—
Resting heart rate	Between mean midpoint deviation	-1.529	-4.727 to 1.670	0.349	—
Wellness strain	Within load	0.498	0.453 to 0.544	< 0.001	—
Wellness strain	Within midpoint deviation	0.287	0.133 to 0.442	< 0.001	—
Wellness strain	Interaction	0.067	-0.005 to 0.138	0.067	0.109
Wellness strain	Between mean load	0.170	-0.071 to 0.411	0.167	—
Wellness strain	Between mean midpoint deviation	-1.192	-1.904 to -0.481	0.001	—
CMJ height	Within load	-0.293	-0.329 to -0.257	< 0.001	—
CMJ height	Within midpoint deviation	-0.228	-0.354 to -0.102	< 0.001	—
CMJ height	Interaction	-0.038	-0.097 to 0.020	0.199	0.199
CMJ height	Between mean load	-2.197	-4.927 to 0.533	0.115	—
CMJ height	Between mean midpoint deviation	-7.124	-15.176 to 0.927	0.083	—

Within-load estimates are per 100 AU; within-midpoint estimates are per 30 min; interaction estimates are per 100 AU × 30 min. Between effects use participant means on the same scales. *FDR adjustment was applied only to the three interaction tests. CMJ, countermovement jump; FDR, false discovery rate.

### 3.4 Sensitivity analyses and diagnostics

The primary interaction remained after adjustment for prior-morning lnRMSSD (β = -0.0096, 95% CI -0.0147 to -0.0046; p < 0.001; 1,063 observations from 54 participants) and after adjustment for sport category (β = -0.0100, 95% CI -0.0153 to -0.0048; p < 0.001). The reduction from 1,232 to 1,063 observations reflected unavailable lagged lnRMSSD: 45 otherwise eligible records occurred on study day 1, and 124 followed a morning without valid HRV. In the exploratory signed-midpoint model, the load-by-direction interaction was not detected (β = 0.0008 per 100 AU × 30 min later shift, 95% CI -0.0024 to 0.0039; p = 0.632), providing no evidence that later versus earlier shifts differentially moderated the load–lnRMSSD association.

The interaction was also similar under autoregressive GEE (β = -0.0124, 95% CI -0.0178 to -0.0071; p < 0.001), in the high-adherence subset (β = -0.0114, 95% CI -0.0189 to -0.0040; p = 0.003), after excluding 13 mornings following sessions ending after 22:00 (β = -0.0100, 95% CI -0.0153 to -0.0047; p < 0.001), after adjustment for sleep duration and efficiency (β = -0.0099, 95% CI -0.0151 to -0.0047; p < 0.001), and with a random-intercept-only model (β = -0.0103, 95% CI -0.0155 to -0.0050; p < 0.001). The unweighted and inverse-probability-weighted independence GEE estimates were similar to each other but imprecise (unweighted β = -0.0080, 95% CI -0.0207 to 0.0048; p = 0.222; weighted β = -0.0081, 95% CI -0.0209 to 0.0047; p = 0.217).

Valid paired sleep–HRV availability was 79.2% among female participants and 81.8% among male participants and ranged from 60.7% to 92.9% across participants. The primary optimizer converged but emitted a boundary warning; random-load-slope variance was 0.000054. The similar random-intercept-only estimate therefore supports the stability of the fixed interaction estimate. Residual skewness was 0.11, excess kurtosis 0.40, residual lag-1 correlation 0.23, and the Spearman correlation between absolute residuals and fitted values 0.025. The autoregressive GEE working-correlation estimate was 0.77.

## 4 Discussion

Greater-than-usual prior-day load was associated with lower next-morning lnRMSSD at a participant’s mean midpoint deviation, and greater-than-usual midpoint deviation was associated with lower lnRMSSD at mean load. Beyond these main effects, the load–lnRMSSD association was moderated by sleep-timing irregularity: the estimated association became more negative as absolute midpoint deviation increased. This pattern is consistent with prior evidence that acute training produces small average HRV changes in free-living conditions and that individual HRV responses vary substantially across athletes and training states (Altini and Plews, 2021; Plews et al., 2017). It extends that work by identifying atypical sleep timing as one contextual correlate of the day-level load–HRV association. In monitoring terms, the same deviation in training load did not have a uniform next-morning association: its inverse association with lnRMSSD was stronger after sleep occurred farther from the participant’s usual timing.

A lower within-participant lnRMSSD reflects lower short-term vagally mediated beat-to-beat variation under the study’s morning conditions. At the within-person level, a transient reduction from an athlete’s usual value can follow acute training, illness, alcohol intake, or sleep deprivation and may indicate short-term autonomic perturbation (Altini and Plews, 2021; Zhang et al., 2025). It is not equivalent to maladaptation. Longitudinal training studies show that increases in vagal HRV often accompany improved performance, but increases can also occur during overreaching, while elite athletes may improve despite decreases in HRV (Plews et al., 2013; Bellenger et al., 2016). At the between-person level, endurance athletes commonly show higher resting HRV than non-athletes, yet stable differences in fitness, age, sex, and measurement conditions limit the meaning of cross-sectional comparisons for individual readiness (Lundstrom et al., 2023). Thus, both lower and higher lnRMSSD can accompany favorable or unfavorable states, depending on the comparison level, time scale, training phase, and concurrent performance or wellness. The present outcome should therefore be interpreted as a next-morning autonomic correlate, not as a direct assay of recovery or adaptation.

Sleep timing is a biologically and behaviorally plausible context, but the mechanism cannot be identified here. Irregular timing can coincide with delayed circadian phase, academic or social scheduling, travel, late training, and altered pre-sleep behavior (Phillips et al., 2017; Walsh et al., 2021). The signed analysis did not indicate that later versus earlier shifts differentially moderated the load association, suggesting that the magnitude of departure from usual timing, rather than direction alone, captured the observed pattern in this sample. Adjustment for sleep duration and efficiency yielded a similar interaction, consistent with timing regularity and sleep quantity representing related but non-equivalent dimensions.

The temporal relationship may also be bidirectional. In junior endurance athletes, sleep, mental strain, and self-selected training load predicted one another across days (Hrozanova et al., 2020). HRV recorded near bedtime has been associated with subsequent sleep continuity in healthy adults (Tramonti Fantozzi et al., 2019), and pre-sleep HRV predicted selected polysomnographic continuity measures in male national-level team athletes (Li et al., 2025). Autonomic status may therefore precede and follow sleep, while morning status may influence that day’s training. Importantly, the primary interaction persisted after adjustment for prior-morning lnRMSSD, but prior perceived status and training decision processes were not fully controlled. Time-varying confounding and feedback remain possible.

Greater-than-usual load and midpoint deviation each had main-effect associations with higher resting heart rate, higher wellness strain, and lower CMJ height. However, there were no interaction effects for these secondary outcomes. Several explanations are plausible. Resting HR and lnRMSSD arise from the same recording but are not interchangeable autonomic summaries; wellness strain is a constructed perceptual composite; and CMJ was sampled only three mornings per week and is subject to smartphone frame-selection error. These differences in construct, sampling density, residual variability, and measurement precision can attenuate interaction estimates. The absence of secondary interactions therefore narrows the result to lnRMSSD and should not be interpreted as evidence that sleep timing has no relevance to other outcomes.

The measurement strategy should be considered as an integrated field-monitoring system. sRPE × duration is practical across training modes but is not a criterion measure of external work and may represent different stimuli across sports. Actigraphy estimates sleep–wake behavior rather than sleep stages and may misclassify quiet wake as sleep (Conley et al., 2019; Halson, 2019). Polar H10 provides valid RR intervals under resting conditions, but spontaneous breathing and day-to-day implementation add variability. Smartphone measurement of CMJ height is generally valid when standardized, yet depends on camera placement and manual frame selection (Balsalobre-Fernández et al., 2015; Gençoğlu et al., 2023). The wellness sum uses common athlete-monitoring domains but was constructed for this study and not independently validated as a scale (Duignan et al., 2020). Consequently, the findings represent within-participant statistical associations among practical indicators; they do not directly quantify sleep physiology, autonomic mechanisms, or neuromuscular recovery.

A further limitation concerns HRV timing. The protocol standardized seated rest, recording duration, and avoidance of caffeine, food, and exercise, but not a fixed clock time or fixed awakening-to-recording interval. Because sleep midpoint and awakening time can shift together and HRV has time-of-day dependence (Vitale et al., 2019), residual confounding by measurement timing may contribute to the moderator association. The available data support adjustment for prior-morning lnRMSSD but not direct separation of clock-time and awakening-interval effects. Future studies should timestamp both awakening and HRV acquisition and model these variables prospectively.

The heterogeneous sport sample also limits inference. Person-mean centering reduces confounding by stable between-participant differences, and sport-category adjustment left the interaction nearly unchanged, but an increment in sRPE-derived load may not represent the same physiological stimulus in endurance, team, and strength–power training. With 54 participants, sport-specific interaction estimates would be unstable. The eligibility criteria captured a cohort of university students training regularly rather than a verified elite, varsity, or competition-defined cohort; findings should not be generalized as sport-specific or elite-athlete evidence.

The 28-day repeated-measures design, separation of within- and between-participant effects, minimum valid-pair threshold, prior-HRV sensitivity analysis, and multiple alternative correlation and random-effects specifications are strengths. Remaining limitations include observational exposure assignment; period-wide calibration of habitual midpoint; unmeasured chronotype, academic stress, travel, menstrual-cycle factors, training type, and prior wellness; broad sport classification; six participants below the valid-pair threshold; a boundary warning for the random load slope; and imprecision in independence GEE. The study cannot establish causal direction, a universal readiness cutoff, a training-cancellation rule, or a sleep-timing prescription.

Future work should use a pre-established or rolling midpoint reference; timestamp awakening, pre-sleep HRV, and morning HRV; collect training mode and decision context; and repeatedly assess prior perceptual status, chronotype, academic stress, travel, and menstrual-cycle factors. Replication across institutions and defined competitive levels should test whether the magnitude and direction of sleep-timing shifts alter load–HRV associations before individualized decision thresholds are considered.

## 5 Conclusion

In this 28-day study, greater-than-usual training load and greater-than-usual absolute sleep-midpoint deviation were each associated with lower next-morning lnRMSSD, and the within-participant load–lnRMSSD association was moderated by sleep-timing irregularity. The interaction persisted after adjustment for prior-morning lnRMSSD and sport category, whereas no analogous interaction was detected for resting heart rate, wellness strain, or CMJ height. The result provides contextual evidence about practical monitoring indicators, but measurement uncertainty, possible timing confounding, and the observational design preclude causal, mechanistic, or diagnostic inferences and prospective training decisions. Although these findings do not support individualized decision-making, they suggest that sleep-timing regularity may be a useful contextual factor to monitor alongside training load in future prospective research.

## Data Availability

The de-identified analysis data and analysis code supporting the findings are available from the corresponding author upon reasonable request.
